# Survival of *Escherichia coli* in Edible Land Snails: Implications for Heliciculture and Public Health

**DOI:** 10.3390/pathogens13030204

**Published:** 2024-02-26

**Authors:** Mary Nkongho Tanyitiku, Graeme Nicholas, Jon J. Sullivan, Igor C. Njombissie Petcheu, Stephen L. W. On

**Affiliations:** 1 Department of Wine, Food and Molecular Biosciences, Lincoln University, Lincoln 7647, New Zealand; 2 Department of Pest-Management and Conservation, Lincoln University, Lincoln 7647, New Zealand; 3 Global Mapping and Environmental Monitoring, Yaounde P.O. Box 755, Cameroon

**Keywords:** edible land snails, microbial pathogens, *E. coli* survival, heliciculture, snail meat consumption, public health

## Abstract

Background: Land snails are considered a delicacy in many countries in Europe and sub-Saharan Africa. However, the interaction of microbial pathogens with land snails may present a public health threat when handling and/or consuming snails. This study examines the survival of *Escherichia coli* in edible land snails in a model system. Methods: Well-studied Shigatoxigenic (STEC) and non-STEC strains were compared. Mature *Helix* spp. were experimentally fed with *E. coli*-inoculated oats for 48 h. The snail feces after inoculation were periodically sampled and cultured for a 30-day period and subjected to microbiological analyses. Results: The average rate of decline of the non-STEC strain CSH-62 in the feces of live snails was significantly (*p* < 0.05) faster than that of STEC ERL 06-2503. In addition, the viable population of *E. coli* ERL 06-2503 significantly (*p* < 0.05) persisted for a longer time in the intestine of land snails than *E. coli* CSH-62. Conclusion: The results showed that the viable population of the *E. coli* strains examined demonstrated first-order kinetics, and their survival (CFU/mL) appeared significantly (*p* < 0.05) dependent on the *E. coli* pathotype. In addition, the continuous enumeration of *E. coli* in snail faeces indicated that land snails could serve as a mode of transmission of microbial pathogens to susceptible hosts, including humans. Further research is recommended to better quantify the direct and indirect health risks of pathogen transmission by edible snails to humans.

## 1. Introduction

Humans consumed snails as early as 10.5 thousand years ago [1]. Land snails were prominent during the era of the Roman Empire, where it was a common aphrodisiac practice to eat snail meat in the courts of the emperor [1,2]. Today, edible land snails are defined by regulation (EC) No. 853/2004 as terrestrial gastropods of the *Helix* and *Achatinidae* families [3]. Although *Helix* spp. (*Helix pomatia Linné*, *Helix aspersa Muller*, *Helix lucorum*) remain a delicacy in many European cuisines, species of the Achatinidae family, specifically *Achatina achatina*, *Achatina fulica*, and *Archachatina marginata*, are popularly consumed in the tropical and savannah zones of Africa [3,4,5,6,7,8]. Its global consumption is currently at 450–500,000 tons per year. Snail meat consumption is in increasing demand due to its ready availability, high-quality protein, balance of omega-3 and omega-6 fatty acids, low fat, high calcium, magnesium, and iron contents [1,9,10,11].

Snail-consuming populations often source edible land snails from non-standard locations, including swamps, ditches, forests, gardens, footpaths, farmlands, and domestic waste areas [7,12,13,14,15]. It has been estimated that only 55,000 tons (13.8%) of the total snails consumed in the world each year are obtained from snail breeding farms [1]. France [11], for example, which is famous for its cuisine of *Helix* spp. (escargots), consumes 44,000 tons per year, yet only 1% are obtained through snail farming [16]. Consumption of snails obtained from unregulated sources may pose public health risks. Significant disease-causing pathogenic microorganisms including *Escherichia coli*, *Campylobacter* spp., *Salmonella* spp., *Listeria* spp., *Yersinia* spp., among others, have been detected in studies of edible land snails in both snail farms and free-living environments [4,5,8,13,15,17,18,19,20]. Notably, 57% of snails sampled in Cameroon were found to harbour Shiga toxin-producing *E. coli* (STEC) [19].

Shigatoxigenic *E*. *coli* is a leading cause of foodborne disease; at least 130 of the 151 serogroups of STEC have been reported to be associated with human illnesses around the world [21]. They are responsible for conditions ranging from mild enteritis to haemorrhagic colitis, which in some cases leads to complications including haemolytic uremic syndrome (HUS) and even death [21]. As a mode of transmission of *E. coli* to humans, some studies have reported the survival of *E. coli* in the faeces of cattle [21,22,23], sheep [22], pigs [22], Canada goose [24], rats, and pigeons [25]. To our knowledge, there are no existing data on the survival characteristics of microbial pathogens in edible land snails.

Considering foodborne disease risks that could arise in snail handling and/or consumption, the purpose of this current study was to evaluate, in controlled conditions, the colonisation potential and survival in feces, of *E. coli* in edible land snails. We also highlight the public health risks of *E. coli* in the faeces of edible land snails and its implications for heliciculture.

## 2. Materials and Methods

### 2.1. E. coli Isolates and Inoculum Pre-Preparation

Two *E. coli* strains were used in this study. *E. coli* ERL 06-2503 is a Shiga-toxigenic variant (serotype O157:H7, containing *stx_2_*, *eae*A and *ehx*A genes) that was found to bind strongly to the bovine intestines in ex vivo experiments [26]. It was obtained from a stock culture collection of the Enteric Reference laboratory, a department of the Institute of Environmental Science and Research (ESR, Christchurch, New Zealand). Another strain, *E. coli* CSH-62, is a non-invasive, non-Shigatoxigenic laboratory-adapted strain that has often been used in transformation experiments [27,28].

Before the inoculation of experimental snails (see Section 2.2), each strain of *E. coli* was recovered from a 40% glycerol stock solution that had been stored at −80 °C. A loopful of each strain of *E. coli* was individually streaked on Tryptone Bile Glucuronic, TBX agar (Oxoid, Hampshire, UK) plates and cultured at 37 °C for 20 h. Serial dilutions of 10^−1^ to 10^−6^ of a single colony in cultured TBX plates were again subcultured in TBX. The presence of *stx1* and *stx2* genes for pathogenic *E. coli* ERL 06-2503 in the cultured plates were verified using a high-fidelity DNA polymerase (repliQa Hifi toughmix: Quantabio, MA, USA), as described in previous PCR protocols [19].

### 2.2. Preparation of Experimental Snails

The snails were prepared for inoculation experiments based on established methods for laboratory snail farming [29] and outdoor snail farming or heliciculture [6,30,31]. In brief, 50 mature land snails (*Helix aspersa*) of reproductive age were collected from vegetable farms in Christchurch, New Zealand, during the summer months of November to January 2019. Snail species were identified by their morphological characteristics; for example, the shells were brown with 4 to 5 whorls and measured 30 to 45 mm [1,30].

**Figure 1:**
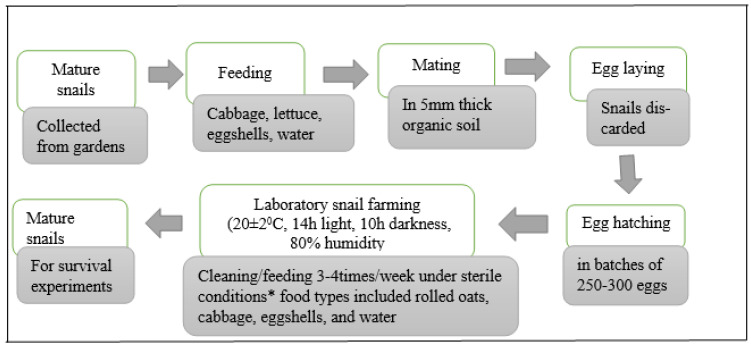
Preparation schedule of snails for bacterial inoculation. * Regular sterilization (121 °C/20 min) of all types of food, materials and equipment used.

Snails were transported to Lincoln University, New Zealand, maintained in optimal growth conditions (20 ± 2 °C, 14 h light, 10 h darkness, 80% humidity), and fed with vegetables (cabbage, lettuce) and eggshells (a calcium carbonate supplement) until egg laying commenced [29,31]. Aseptically, egg batches were embedded into 5 mm thick pre-autoclaved organic soil (Garden box, Christchurch, New Zealand), kept moist in sterile distilled water, and contained in 2-L perforated Sistema containers (Sistema Plastics, Auckland, New Zealand) pre-treated with exposure to ultraviolet light for ≥2 h. Within 18–21 days, juvenile snails emerged from the organic soil and the snails were raised till maturity, that is, 18 months. A summary of the rearing procedure is presented in Figure 1.

### 2.3. Inoculation of Experimental Snails

**Figure 2:**
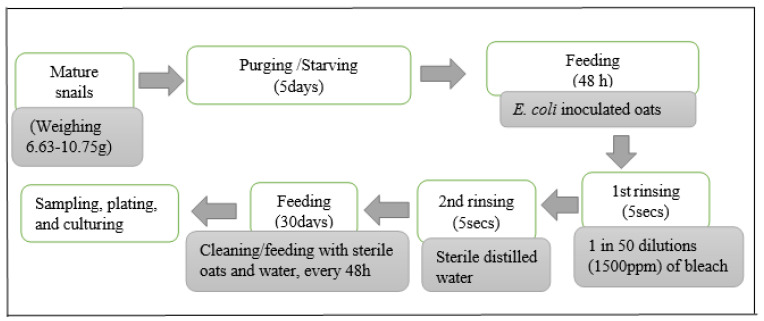
Inoculation schedule of snails for *E. coli* survival experiments.

All inoculation experiments were conducted in a biological safety hood. Bacteria suspensions (1 mL) were transferred into 15 mL Eppendorf tubes containing 1 g of moist rolled oats each. The rolled oats were rehydrated according to the manufacturer’s instructions (Harraways, Dunedin, New Zealand). The inoculant concentration in the rolled oats was such that when initially examined on TBX agar at 36 °C for 20 h, the bacterial colonies were approximately 10^6^ CFU/mL, based on a 10^−3^ dilution of a suspension made to an optical density of 1.0 determined at 600 nm (SmartSpec, Bio-Rad, Hercules, CA, USA). A total of 40 tubes were prepared, thoroughly mixed, and poured into a sterile 2-L perforated Sistema container (Sistema Plastics, Auckland, New Zealand). 40 mature land snails, previously purged with rolled oats for 36 h and then starved for 48 h [29], were then introduced into the mixture of bacteria-rolled oats for 48 h. In principle, this implied that 1 g of inoculated oats corresponded to 1 land snail. Oats used throughout the study had been previously screened for the presence of *E. coli* (using methods described by [32], modified by the use of 5 g samples in the protocol) and tested negative for this organism. The procedure is summarised in Figure 2.

After 48 h of exposure and feeding on inoculated oats, the snails were rinsed for ca. 5 s with 15 mL of 5% sodium hypochlorite (Janola premium bleach, Pental Products Pty Ltd., Melbourne, Australia) and then 15 mL of sterile distilled water to achieve surface decontamination. Preliminary experiments with snails intentionally contaminated with *E. coli* demonstrated that this protocol was effective in snail surface decontamination when rinse water was cultured on Nutrient agar no. 2 (Oxoid, Hampshire, UK) at 37 °C for 18 h. Snails remained viable after this treatment.

### 2.4. E. coli Survival Experiments

After the brief rinsing with 5% sodium hypochlorite and distilled water, the 40 snails from each *E. coli* strain inoculation were used as the starting samples for the 30-day survival experiments (Figure 2). Snails were then fed with 40 g of sterile moist rolled oats, and periodically every 48 h, their faeces were collected at the point of cleaning and feeding with fresh rolled oats. The enumeration of *E. coli* in snail faeces was conducted based on Silva, Marta [32], with slight modifications. 5 g of pooled snail faeces from each inoculated group in one “Sistema” container were thoroughly vortexed in 45 mL of Phosphate buffered saline, PBS (10^−1^ dilution), and the suspension was allowed to stand for 30 mins. Subsequent dilutions (10^−1^ to 10^−6^) of the sample were prepared, and 1 mL each of three appropriate dilutions (10^−1^, 10^−2^, 10^−4^) were inoculated into Durham tubes containing 2 mL of *E. coli* (EC) broth. The samples were then incubated at 44.5 ± 0.2 °C/24 h in a shaker incubator (ThermoScientific, Auckland, New Zealand), rotating at 120 rpm.

Using a spread plate method, 50 µL of serial dilutions (10^−1^ to 10^−6^) of EC broth samples were plated on TBX agar. The plates were incubated at 37 °C for 20 h, and 30–250 bacteria colonies were visually counted using a 6X LED illuminated linen tester (Magnifiers, Christchurch, New Zealand). The surviving colonies of *E. coli* were confirmed as blue-green colonies in TBX agar plates [33]. Further *E. coli* confirmation tests for inconclusive colonies were performed using previously described PCR protocols [19]. The same procedure was used to screen for *E. coli* in rolled oats prior to feeding inoculated snails during the entire 30-day survival experiments. It should be noted that 30 days were determined as the maximum period for survival experiment that allowed for adequate faecal sample collection for microbiological analyses, since it was observed that snails barely survived beyond this period under the aforementioned post-inoculated laboratory feeding and growth conditions.

### 2.5. Statistical Analysis

All experiments were carried out in triplicate. The values of CFU/mL for each timepoint were expressed as the mean ± standard deviation of the three separate survival experiments, with the mean values used as the basis for statistical analysis. A commercial spreadsheet software (Microsoft 2010) was used to perform all calculations. R programming (version 4.0.3) was used in the plotting of the best-fit graphs and in performing statistical analyses, that is, Analysis of Variance (ANOVA) and student *t*-tests. The mean number of CFU/mL from each sampling time was calculated and represented as logarithmic transformed data; that is, each calculated CFU/mL was replaced with the transformed value log_10_(x), where x = CFU/mL. The normal distribution of each logarithmic transformed data set was graphically verified (with a confidence level of 95%). Linear regression analysis was used to determine the best relationship between the two *E. coli* strains. The D-values (the time required to reduce the *E. coli* population by 1log cycle) were also determined by taking the negative reciprocal of the slope from the linear regression equations [34].

## 3. Results

**Figure 3:**
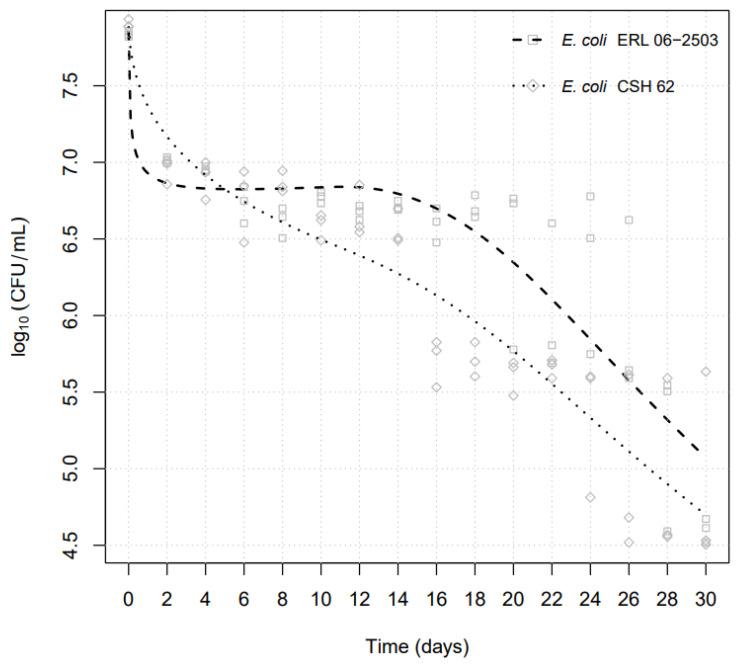
Survival in snails of *E. coli* strains STEC ERL 06-2503 and laboratory-adapted strain CSH 62 during the 30-day examination period, using the “best fit” algorithm in the “R” software environment.

The number of CFU/mL for each strain decreased over the examination period (Supplementary Tables S1–S5). The average rate of decline of the laboratory-adapted *E. coli* strain CSH-62 in the faeces of live snails was significantly (*p* < 0.05) faster than the pathogenic (Shiga-toxigenic) *E. coli* ERL 06-2503. Furthermore, the viable population of the Shiga-toxigenic *E. coli* strain ERL 06-2503 persisted significantly (*p* < 0.05) for a longer time in the intestinal tract of land snails than that of the laboratory-adapted *E. coli* CSH-62. These trends are clearly seen in the graph using the “best fit” algorithm in the “R” software environment (Version R 4.2.0) (Figure 3).

Strain ERL 06-2503 showed a relatively rapid decrease to 6.78 log_10_ CFUmL^−1^ within the first two days but was constant for the next 14 days before a marked decrease at day 28; in contrast, *E. coli* strain CSH-62 decreased steadily throughout the experiment (Figure 3). For both strains, viable bacteria remained detectable up to the end of the sampling period, that is, approximately 165,000 CFU/mL for *E*. *coli* CSH-62 and 403,000 CFU/mL for *E. coli* ERL 06-2503. The mean decline in the viable population demonstrated first-order kinetics. With no clear evidence of shoulders or tails, this permitted the calculation of the decimal reduction times (D-values) from the linear parts of the survival curves [22,34].

*E. coli* CSH-62 displayed three timepoints at which a 1-log decrease in its viable population was evident (Table 1). In the first two days, a 0.95 log_10_ CFUmL^−1^ decrease in viable counts was observed. The calculated D value (time for a 1 log decrease in *E. coli*) for this initial time period (0 to 2 days) was 1.59 days. An additional decline of 1.28 log_10_ CFUmL^−1^ in viable bacteria occurred during the next 18 days. The calculated D-value for this second phase (days 2 to 18) was 14.62 days. Finally, the third decrease in the viable count of 1.07 log_10_ CFUmL^−1^ for the remaining 30 days of the study occurred between day 18 and day 26. Its D-value was 25.36 days.

However, corresponding analysis of the results for STEC strain ERL 06-2503 revealed only two timepoints at which a 1-log decrease was seen (Table 1). An initial decrease in viable counts of 1.11 log_10_ CFUmL^−1^ was observed on day 2 after inoculation. The calculated D-value for day 0 to 2 was 1.38 days. The second decrease of 1.03 log_10_ CFUmL^−1^ in viable count was observed on day 22. The calculated D-value (day 2 to 22) was 18.08 days. From day 22 to 30, we obtained a D-value of 28.96 days, of which a further 1.03 log_10_ CFUmL^−1^ of viable bacteria was observed on day 30 (the last day of sampling).

## 4. Discussion

Snail farmers all over the world are experimenting with different types of indoor and outdoor snail production systems. The challenges in adapting well-defined snail farming systems are due to the ability of snails to adversely respond to changes in climatic conditions [6,31]. In both farming systems, it is well known that land snails, such as African giant snails, are hosts to many parasites and bacterial pathogens [4,5,8,13,15,17,18,19,20,35]. Indeed, our previous work [19] detected at least one enteric pathogen in every snail sample examined, with STEC found in 57% of the samples examined. In this research, we studied *E. coli* interactions with land snails, *Helix* spp., to gain an understanding of the colonisation dynamics and infectious potential of this relationship. Our results show that *E. coli* can survive in edible land snails for at least a month and that pathogenic variants such as STEC may survive for longer.

Land snails are known to occupy various environments and are susceptible to *E. coli* contamination due to (1) their free-living nature, (2) their close contact with the soil and (3) their random feeding patterns [4,5,8,19]. Previous research has also detected different strains of pathogenic *E. coli* in the faeces of healthy animals that were raised in indoor and grassland systems [22,23]. For example, to establish the transmission pattern of *E. coli* O157: H7 between cattle groups, Scott and McGee [36] reported that a total of 8.7% (6/69) of cattle shed *E. coli* O157: H7 in the first month, with faecal shedding increasing to 52% (36/69) in the third month after infection. Kudva and Blanch [23] recovered *E. coli* O157: H7 in the range of <10^2^ to <10^6^ CFU/g in the manure of inoculated sheep, further demonstrating that *E. coli* could survive for 21 months in bovine faeces, despite fluctuating environmental conditions. Furthermore, Avery and Moore [22] demonstrated that *E. coli* from the faeces of indoor cattle, sheep and pigs can survive on grass for at least 5–6 months. Under such conditions, host animals are continually re-exposed to strains from the environment and possess body temperatures favourable to *E. coli* multiplication. Snails are poikilothermic and, in our experiments, exposed only once to the inoculant. Despite these conditions, both pathogenic and laboratory-adapted strains of *E. coli* were detected for 30 days. This suggests that the faeces of infected snails in the environment could act as a significant reservoir for the transmission of *E. coli* to humans and/or animals. Moreover, our study suggests that current helicicultural practices expose snail handlers, and subsequently consumers, to foodborne pathogens.

The presence of *E. coli* in the faeces of snails further revealed that snails as intermediate hosts and/or the consumption of raw or undercooked snail meat may not necessarily be the only route to foodborne illnesses associated with land snails. For example, snails are known to leave their slime and faeces on locally produced green leafy vegetables in gardens, which is an example of this research’s initial snail harvesting location. Humans could directly ingest these bacterial pathogens from the faeces of snails present in their daily gardening. For example, in 2021, *E. coli* O157: H7 foodborne outbreaks were reported in baby spinach, leafy greens, and several unknown food sources in the United States [37]. Mukherjee et al. [38] reported a sporadic case of *E. coli* O157 infection in a child who acquired *E. coli* O157: H7 from garden soil that had been previously fertilized with manure from cattle in Minnesota, USA. Through PCR and pulsed-field gel electrophoresis techniques, it was demonstrated that *E. coli* O157:H7 strains can survive on manure-amended soil for more than 2 months [38].

Using the non-invasive laboratory-adapted *E. coli* strain CSH-62 and Shiga-toxigenic pathogenic *E. coli* strain ERL 06-2503, this research has demonstrated that the outcome of *E. coli* interactions with land snails may be significantly (*p* < 0.05) strain dependent. The STEC strain used was specifically chosen for its ability to robustly colonize an ex vivo bovine intestinal model; more robustly, in fact, than other strains studied [26]. Strain variance was strongly evidenced in our study. Strain CSH-62 demonstrated an approximate 3 log_10_ CFUmL^−1^ decline in its viable population, while the pathogenic *E. coli* strain ERL 06-2503 demonstrated 2 log_10_ CFUmL^−1^ reduction in its viable population. Similar results have been reported [39], where invasive *E. coli* K1 had the ability to penetrate and remain viable in the free-living protozoan *Acanthamoeba*, while non-invasive *E. coli* K-12 was progressively killed.

Although it is unclear how both *E. coli* strains maintained a somewhat prolonged survival in land snails, their continued persistence in edible land snails represents significant risks to public health. Previous recommendations [31,40] indicate that the purging/starvation of land snails for 3–5 days prior to human consumption will accelerate the process of ridding the snail digestive system of soil, food stuffs and microorganisms. However, this is contrary to our findings, as both pathogenic and laboratory-adapted *E. coli* strains were actively viable beyond day 5 after inoculation and during the entire period whereby the sole nutrition source was soaked oats, a commonly used purging agent. Our results provide evidence that land snails may host both pathogenic and non-pathogenic *E. coli* for prolonged periods (30 days), which may be transmitted to susceptible hosts, including humans. More research is required to understand the fate of *E. coli* after this experimental research period, the survival mechanisms of the different *E. coli* serotypes in the guts of edible land snails, and the spectrum of survival behaviours from additional strains and pathovars of importance to human health.

## 5. Conclusions

In summary, this study revealed that upon ingestion and continuous shedding in the faeces of snails, *E. coli* could survive in edible land snails for more than a month. Although the results indicate that the viable populations of *E. coli* declined significantly within the 30-day experimental period, the ability of *E. coli* to survive throughout the study under controlled laboratory conditions precluding reinfection is of great significance for helicicultural practices and public health, especially in regions where the carriage of pathogenic Shigatoxigenic strains is so prevalent [19] and where snails are frequently consumed as a source of nutrition. We accept that our results, based on only two (albeit well characterised) strains, may be regarded as preliminary, an unfortunate casualty of issues concerning access to laboratories during the COVID-19 lockdown period in New Zealand. Nonetheless, we feel the data further illustrate differences between *E. coli* strains in their host colonisation ability, as identified before in a cattle gut model system [26]. We also hope that this inaugural study of *E. coli* colonisation and survival in edible snails can inform better helicicultural practices to reduce the risks of foodborne illness. Clearly, there are implications for existing practices where snails are sourced from native, uncontrolled conditions, and there may well be advantages in this respect in encouraging a more widespread practice of snail rearing under more hygienic and controlled conditions. The need for legislation to formalise such interventions is open to conjecture.

## Figures and Tables

**Table 1:** Calculated D-values of *E. coli* strains examined within the 30-day period at timepoints showing a ≥1-log decrease in colony forming units.

*E. coli* Isolates	Day of ~1log Cycle	log_10_ CFUmL^−1^	D-Value (Days)
*E. coli* CSH-62	2	6.952	1.59
	18	5.675	14.62
	26	4.604	25.36
*E. coli* ERL 06-2503	2	6.795	1.38
	22	5.765	18.08

## Data Availability

All applicable data are given in the Supplementary Tables S1–S5.

## Supplementary Materials

The following supporting information can be downloaded at: https://www.mdpi.com/article/10.3390/pathogens13030204/s1, Table S1: Reformatted data; Table S2: Means SDs CSH62; Table S3: Means SDs STEC; Table S4: Raw data; Table S5: Corrected data.
